# Association between inflammatory bowel disease and the risk of parenteral malignancies: A two-sample Mendelian randomization study

**DOI:** 10.1016/j.clinsp.2024.100421

**Published:** 2024-06-28

**Authors:** Peizhu Su, Yilin Wang, Huiwen Huang, Qinghua Lu, Qinyan Wu, Zhaotao Li

**Affiliations:** Department of Digestive Disease, The First People's Hospital of Foshan City, Guangdong, P.R. China

**Keywords:** Inflammatory bowel disease, Parenteral malignancies, Skin cancer, Mendelian randomization study, Causal relationship

## Abstract

**Aim:**

Using Mendelian Randomization (MR) analysis to investigate the potential causal association between Inflammatory Bowel Disease (IBD) and the occurrence of parenteral malignancies, in order to provide some reference for the parenteral malignancy prevention in patients with IBD.

**Methods:**

This was a two-sample MR study based on independent genetic variants strongly linked to IBD selected from the Genome-Wide Association Study (GWAS) meta-analysis carried out by the International Inflammatory Bowel Disease Genetics Consortium (IIBDGC). Parenteral malignancy cases and controls were obtained from the FinnGen consortium and the UK Biobank (UKB) release data. Inverse Variance Weighted (IVW), weighted median, MR-Egger, and strength test (F) were utilized to explore the causal association of IBD with parenteral malignancies. In addition, Cochran's Q statistic was performed to quantify the heterogeneity of Instrumental Variables (IVs).

**Results:**

The estimates of IVW showed that patients with IBD had higher odds of diffuse large B-cell lymphoma (OR = 1.2450, 95% CI: 1.0311‒1.5034). UC had potential causal associations with non-melanoma skin cancer (all p < 0.05), melanoma (OR = 1.0280, 95% CI: 0.9860‒1.0718), and skin cancer (OR = 1.0004, 95% CI: 1.0001‒1.0006). Also, having CD was associated with higher odds of non-melanoma skin cancer (all p < 0.05) and skin cancer (OR = 1.0287, 95% CI: 1.0022‒1.0559). In addition, results of pleiotropy and heterogeneity tests indicated these results are relatively robust.

**Conclusions:**

IBD has potential causal associations with diffuse large B-cell lymphoma and skin cancers, which may provide some information on the prevention of parenteral malignancies in patients with IBD. Moreover, further studies are needed to explore the specific mechanisms of the effect of IBD on skin cancers.

## Highlights

• To provide some reference for parenteral malignancy prevention in patients with IBD.
• IBD was potentially associated with diffuse large B-cell lymphoma and skin cancers.
• Some information on preventing parenteral malignancies in IBD was provided.
• Further studies are needed to explore mechanisms of the effect of IBD on skin cancers.

## Introduction

Inflammatory Bowel Disease (IBD) is an immune-mediated intestinal tract disease, including Crohn's Disease (CD) and Ulcerative Colitis (UC), which is related to the complex interaction between the genetic, environmental, gut microbiome, and immune factors.^1^ Malignancy is now the second leading cause of mortality in patients with IBD.^2^ Due to the influencing of chronic inflammation in the gut, patients with IBD are more likely to develop Colorectal Cancer (CRC) and other intestinal malignancies, whereas the association between IBD and parenteral malignancies is unclear.^3^ Studies have shown that the majority of patients with IBD had parenteral malignancies, and the incidence was gradually increasing.^4^, ^5^, ^6^ With the widespread use of immunosuppressive therapy in IBD, the impaired immune environment of patients may weaken their defense against tumors, so systemic inflammation and long-term immunosuppression caused by IBD may lead to an increased risk of parenteral malignancy.^5^ In addition, the comorbidities and parenteral symptoms of IBD may increase the risk of parenteral malignancy as well.^7^ At present, some observational studies and related meta-studies have reported the risk of specific site malignant tumors in patients with IBD, but no consistent results have been obtained.^8^^,^^9^ Moreover, traditional epidemiological studies are susceptible to confounding factors and causal inversion, and the true relationship between IBD and the risk of parenteral malignancies is unrevealed.

Mendelian Randomization (MR) uses genetic variation as an instrumental variable for exposure to investigate causal associations between exposures and diseases.^10^ Because of Mendel's law of segregation and independent classification, the results of MR analyses are less susceptible to confounding bias than those of traditional observational epidemiological studies.^11^ Also, since the genetic code is not influenced by environmental factors or preclinical diseases, and is less susceptible to bias caused by reverse causation. Therefore, MR analysis is a good choice for the exploration on the causality of IBD with the occurrence of parenteral malignancies. In the latest MR study conducted by Gao et al.^12^ on the causal relationship between IBD and extracolonic cancers in different sites, they found IBD may play a risk role in the development of both the oral cavity and breast cancer. However, there are some unresolved problems with the robustness of the results in Gao's study, which are associated with the horizontal pleiotropy and heterogeneity (where the physical distance ≥ 5000 kb and the Linkage Disequilibrium [LD] r^2^ < 0.01).

Herein, based on the previous study, the authors conducted a two‐sample MR study to investigate the causal association between IBD to parenteral malignancies, with a stricter LD threshold (r^2^ = 0.001 and clumping distance of 10,000 kb). In addition, the authors calculated the power of the Inverse Variance Weighted (IVW) test, in order to improve the robustness of positive results. The authors hope the present findings may further verify the causal association between IBD and parenteral malignancies.

## Methods

### Data sources

**Fig. 1:**
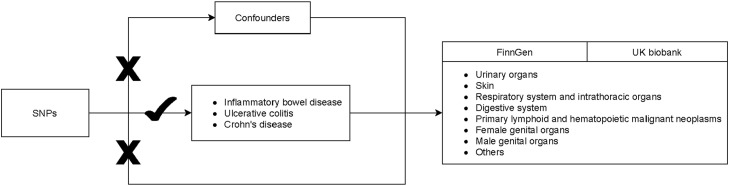
Flowchart of the study procedure.

Figure 1 shows the study procedure. In this two-sample MR analysis, information on IBD and parenteral malignancies were extracted from the corresponding Genome-Wide Association Studies (GWASs): https://gwas.mrcieu.ac.uk/. Genetic variants of the IBD were extracted from the International Inflammatory Bowel Disease Genetics Consortium (IIBDGC). IIBDGC is the largest global IBD genetics database, in which the authors obtained SNPs from the European populations. Parenteral malignancies cases and controls were obtained from the FinnGen consortium (https://finngen.gitbook.io/documentation/) as well as from the UK Biobank (UKB) (https://www.ukbiobank.ac.uk/). More information on study exposure and outcomes are shown in Table 1. Data in the current study are publicly available and de-identified. Each GWAS involved has obtained informed consent from participants and had ethical approval from their respective institutions. Therefore, no ethical approval from the Institutional Review Board (IRB) of The First People's Hospital of Foshan City was required. This Study follows the STROBE Statement.

**Table 1:** Information of the data source for IBD and parenteral malignancies.

Variables	GWAS ID	
Exposures		
IBD	ieu-a-294	
UC	ieu-a-32	
CD	ieu-a-10	
Outcomes		
Breast cancer	finn-b-C3_BREAST	ukb-b-16890
Glioma	finn-b-C3_GBM	
Brain cancer	finn-b-C3_BRAIN	
Meningioma	finn-b-C3_MENINGES	
Thyroid cancer	finn-b-C3_THYROID_GLAND	
Oral and pharyngeal cancer	finn-b-C3_LIP_ORAL_PHARYNX	ieu-b-4961
Urinary organs cancer	finn-b-C3_URINARY_TRACT	ukb-d-C3_URINARY_TRACT
Bladder cancer	finn-b-C3_BLADDER	ukb-d-C67
Kidney cancer (except renal pelvis)	finn-b-C3_KIDNEY_NOTRENALPELVIS	ukb-b-1316
Skin cancer	finn-b-C3_SKIN	ukb-b-12339
Melanoma	finn-b-C3_MELANOMA_SKIN	ieu-b-4969
Non-melanoma skin cancer	finn-b-C3_OTHER_SKIN	ieu-b-4959
Respiratory system cancers	finn-b-C3_RESPIRATORY_INTRATHORACIC	ukb-d-C3_RESPIRATORY_INTRATHORACIC
Bronchogenic carcinoma and lung cancer	finn-b-C3_BRONCHUS_LUNG	ukb-d-C34
Non-small cell lung cancer	finn-b-C3_LUNG_NONSMALL	
Small cell lung cancer	finn-b-C3_SCLC	
Gastric carcinoma	finn-b-C3_STOMACH	
Esophagus cancer	finn-b-C3_OESOPHAGUS	
Liver cancer	finn-b-C3_LIVER_INTRAHEPATIC_BILE_DUCTS	
Pancreatic cancer	finn-b-C3_PANCREAS	
Female genital organs cancers	finn-b-C3_FEMALE_GENITAL	ukb-d-C_FEMALE_GENITAL
Cervical cancer	finn-b-C3_CERVIX_UTERI	ukb-b-8777
Uterine cancer	finn-b-C3_CORPUS_UTERI	ukb-d-C3_CORPUS_UTERI
Ovarian cancer	finn-b-C3_OVARY	ukb-b-18157
Hematopoietic system cancer	finn-b-CD2_PRIMARY_LYMPHOID_HEMATOPOIETIC	leukaemiaukb-d-C3_PRIMARY_LYMPHOID_HEMATOPOIETIC
	finn-b-CD2_HODGKIN_LYMPHOMA	
Diffuse large B-cell lymphoma	finn-b-C3_DLBCL	
Follicular lymphoma	finn-b-CD2_FOLLICULAR_LYMPHOMA	
Mature T/NK-cell lymphomas	finn-b-CD2_TNK_LYMPHOMA	
Lymphoid leukaemia	finn-b-CD2_LYMPHOID_LEUKAEMIA	
Multiple myeloma and malignant plasma cell cancers	finn-b-CD2_MULTIPLE_MYELOMA_PLASMA_CELL	ieu-b-4957
Male genital organs cancers	finn-b-C3_MALE_GENITAL	ukb-d-C_MALE_GENITAL
Prostatic cancer	finn-b-C3_PROSTATE	ukb-b-2160

IBD, Inflammatory Bowel Disease; GWAS, Genome Wide Association Study, UC, Ulcerative Colitis; CD, Crohn's Disease.

### Single nucleotide polymorphisms selection

SNPs that significantly linked to IBD were selected as potential IVs. The threshold of p < 5.0 × 10^−8^ was used to select the IVs. The authors removed SNPs that Minor Allele Frequency (MAF) ≤ 0.01. The LD threshold and the clumping distance were respectively r^2^ = 0.001 and 10,000 kb. MR-Egger regression test was applied to monitor potential horizontal pleiotropy effect, that is the confounding effect resulted from other diseases, and could violate the MR analysis’ second assumption.^13^ The significant intercept item in MR-Egger represents there is a pleiotropy. Besides, due to the principle of MR is to ensure a same allele corresponds effects between SNPs and exposure/outcome, palindromic SNPs need to be deleted.

### The assumptions of MR analysis

The MR analysis must conform to three important assumptions to minimize the impact of bias on the results. Firstly, the IVs must be independent of confounding factors related to exposure and outcome. Secondly, IVs should be significantly associated with the exposure. The authors estimated the relationship strength of IBD with IVs with the formulas: r^2^ = 2 * minor allele frequency (MAF) * (1 - MAF) * β * β / SD^2^; F = ((Sample size - numbers of IVs - 1) / numbers of IVs) * (r^2^ / (1 - r^2^)), in which β was the regression coefficient for IBD and IVs and SD represented standard deviation. F < 10 is considered as there is a weak association between IVs and exposure. Thirdly, IVs only affect outcomes through exposure, namely, no horizontal pleiotropy effect of IVs on the outcome.

### Statistical analysis

Study statistical analysis was performed using R version 4.2.0 (Institute for Statistics and Mathematics, Vienna, Austria). MR analysis on potential causality from IBD to parenteral malignancies was explored through the R package “TwoSampleMR”; p < 0.05 means the evidence for potential causal effect was statistically significant. The calculation for the causal effect values used the IVW test, which is the primary method to acquire unbiased estimates when no horizontal pleiotropy exists. In addition, weighted-median method relatively provides a robust and consistent estimate of the effect even if nearly 50% of genetic variants were invalid instruments. Odds Ratios (ORs) and 95% Confidence Intervals (CIs) were used to express the effect size.

Test for heterogeneity was used Cochrane's Q method, IVs with p < 0.05 were considered as non-heterogeneous.^14^ MR-Egger regression's intercept examined the presence of potential pleiotropy in IVs, and p > 0.05 was recognized as no horizontal pleiotropy. Moreover, the authors calculated the test power of IVW method using the calculation tool on the webpage: https://shiny.cnsgenomics.com/mRnd/.

## Results

### Instrumental variables selection

The authors respectively identified 7,495 SNPs as IVs for IBD, 6,616 SNPs as IVs for UC, and 2,860 SNPs as IVs for CD. After deleting LD and dropping all palindromic SNPs, the final numbers of SNPs in different outcomes are shown in Table 2. Then the authors evaluated the horizontal pleiotropy effect of both exposures and outcomes. For IBD, UC, and CD, none of the IVs in the analyses had horizontal pleiotropy or heterogeneity after removing pleiotropic SNPs that were identified respectively by the MR-Egger intercept test and MR-Egger Q test (all p > 0.05).

**Table 2:** SNPs selection and test for horizontal pleiotropy, strength, and heterogeneity.

**Exposures**	**Phenotypes**	**Outcomes**	**Selected SNPs (p < 5×10^−8^)**	**Omitted LD SNPs**	**Drop all palindromic SNPs**	**Horizontal pleiotropic**	**Heterogeneity**		**Strength**
						**MR-Egger intercept test, p**	**MR-Egger Q, p**	**IVW, p**	**F-value, R^2^**
IBD	Others	Breast cancer	7495	132	125	-0.01, 0.09	153.83, 0.01	157.76, 0.01	31.858,0.061
		Glioma	7495	132	127	-0.02, 0.56	90.99, 0.96	91.33, 0.96	32.14,0.062
		Brain cancer	7495	132	125	-0.01, 0.41	94.63, 0.91	95.32, 0.91	32.672,0.062
		Meningioma	7495	132	129	-0.01, 0.72	113.55, 0.60	113.68, 0.62	32.288,0.064
		Thyroid cancer	7495	132	127	0.01, 0.55	91.72, 0.96	92.08, 0.96	32.733,0.063
		Oral and pharyngeal cancer	7495	132	127	0.02, 0.57	110.19, 0.63	110.51, 0.65	32.48,0.063
	Urinary system	Urinary organs cancer	7495	132	128	-0.00, 0.74	112.17, 0.61	112.28, 0.63	32.457,0.063
		Bladder cancer	7495	132	125	-0.01, 0.25	87.72, 0.97	89.07, 0.97	32.182,0.061
		Kidney cancer (except renal pelvis)	7495	132	125	0.01, 0.35	119.53, 0.39	120.43, 0.40	31.793,0.061
	Skin	Skin cancer	7495	132	120	0.00, 0.98	110.60, 0.44	110.60, 0.47	33.015,0.06
		Melanoma	7495	132	129	0.00, 0.98	97.14, 0.92	97.14, 0.93	32.462,0.064
		Non-melanoma skin cancer	7495	132	120	0.00, 0.98	110.64, 0.44	110.64, 0.47	33.015,0.06
	Respiratory system and intrathoracic organs	Respiratory system cancers	7495	132	123	0.00, 0.63	99.02, 0.80	99.26, 0.82	31.488,0.059
		Bronchogenic carcinoma and lung cancer	7495	132	121	-0.00, 0.65	90.58, 0.91	90.78, 0.92	32.045,0.059
		Non-small cell lung cancer	7495	132	127	-0.01, 0.12	116.77, 0.46	119.28, 0.42	32.691,0.063
		Small cell lung cancer	7495	132	127	0.02, 0.37	101.70, 0.83	102.51, 0.83	32.618,0.063
	Digestive system	Gastric carcinoma	7495	132	130	-0.00, 0.91	104.66, 0.82	104.68, 0.84	32.331,0.064
		Esophagus cancer	7495	132	129	-0.02, 0.42	106.40, 0.77	107.06, 0.78	31.998,0.063
		Liver cancer	7495	132	125	0.00, 0.9	92.95, 0.93	92.97, 0.93	32.157,0.061
		Pancreatic cancer	7495	132	130	-0.00, 0.83	112.04, 0.66	112.09, 0.68	32.331,0.064
	Female genital organs	Female genital organs cancers	7495	132	127	0.01, 0.23	97.09, 0.90	98.57, 0.89	32.503,0.063
		Cervical cancer	7495	132	126	0.01, 0.44	98.86, 0.86	99.46, 0.86	32.099,0.062
		Uterine cancer	7495	132	125	0.01, 0.55	93.39, 0.92	93.74, 0.93	32.978,0.063
		Ovarian cancer	7495	132	128	-0.01, 0.56	103.07, 0.82	103.40, 0.83	32.071,0.063
	Primary lymphoid and hematopoietic system	Hematopoietic system cancer	7495	132	127	0.01, 0.38	147.46, 0.03	148.44, 0.03	32.846,0.064
		Hodgkin lymphoma	7495	132	122	0.00, 0.92	100.10, 0.78	100.11, 0.80	32.908,0.061
		Diffuse large B-cell lymphoma	7495	132	129	-0.04, 0.15	119.13, 0.45	121.27, 0.42	32.123,0.063
		Follicular lymphoma	7495	132	126	-0.01, 0.41	128.21, 0.19	128.98, 0.19	31.277,0.06
		Mature T/NK-cell lymphomas	7495	132	129	-0.02, 0.52	106.17, 0.77	106.58, 0.79	31.928,0.063
		Lymphoid leukaemia	7495	132	129	0.03, 0.06	105.33, 0.81	108.81, 0.76	32.582,0.064
		Multiple myeloma and malignant plasma cell cancers	7495	132	126	0.01, 0.72	106.39, 0.70	106.52, 0.72	32.018,0.062
	Male genital organs	Male genital organs cancers	7495	132	121	0.01, 0.14	102.09, 0.69	104.25, 0.66	33.259,0.061
		Prostatic cancer	7495	132	122	0.00, 0.46	114.96, 0.38	115.54, 0.39	32.938,0.061
UC	Others	Breast cancer	6616	38	35	0.00, 0.75	33.50, 0.18	33.63, 0.21	17.389,0.022
		Glioma	6616	38	35	0.07, 0.43	14.34, 0.98	14.99, 0.99	20.099,0.026
		Brain cancer	6616	38	35	0.03, 0.46	23.78, 0.64	24.33, 0.66	19.171,0.024
		Meningioma	6616	38	35	0.03, 0.34	17.47, 0.92	18.41, 0.92	19.215,0.025
		Thyroid cancer	6616	38	35	0.01, 0.68	22.69, 0.75	22.87, 0.78	20.099,0.026
		Oral and pharyngeal cancer	6616	38	36	0.05, 0.53	28.48, 0.44	28.90, 0.47	19.54,0.026
	Urinary system	Urinary organs cancer	6616	38	36	-0.02, 0.25	23.90, 0.69	25.30, 0.66	19.54,0.026
		Bladder cancer	6616	38	35	-0.03, 0.25	11.50, 1.00	12.90, 1.00	20.099,0.026
		Kidney cancer (except renal pelvis)	6616	38	36	-0.00, 0.93	33.08, 0.23	33.09, 0.27	19.54,0.026
	Skin	Skin cancer	6616	38	34	-0.02, 0.05	19.53, 0.81	23.87, 0.64	17.512,0.022
		Melanoma	6616	38	36	-0.10, 0.27	23.05, 0.73	24.30, 0.71	19.54,0.026
		Non-melanoma skin cancer	6616	38	34	-0.02, 0.05	19.54, 0.81	23.84, 0.64	17.512,0.022
	Respiratory system and intrathoracic organs	Respiratory system cancers	6616	38	34	-0.00, 0.86	27.11, 0.46	27.14, 0.51	19.971,0.025
		Bronchogenic carcinoma and lung cancer	6616	38	35	-0.02, 0.45	30.18, 0.35	30.80, 0.37	20.099,0.026
		Non-small cell lung cancer	6616	38	36	-0.04, 0.11	27.10, 0.51	29.91, 0.42	19.54,0.026
		Small cell lung cancer	6616	38	35	-0.02, 0.81	16.53, 0.94	16.59, 0.96	19.726,0.025
	Digestive system	Gastric carcinoma	6616	38	36	0.00, 0.93	29.14, 0.41	29.15, 0.46	19.54,0.026
		Esophagus cancer	6616	38	35	-0.08, 0.26	34.54, 0.15	36.26, 0.14	19.47,0.025
		Liver cancer	6616	38	35	-0.06, 0.21	22.87, 0.69	24.52, 0.65	19.197,0.024
		Pancreatic cancer	6616	38	36	-0.06, 0.11	21.17, 0.82	23.92, 0.73	19.54,0.026
	Female genital organs	Female genital organs cancers	6616	38	36	0.02, 0.14	29.95, 0.37	32.48, 0.30	19.54,0.026
		Cervical cancer	6616	38	33	0.03, 0.25	25.74, 0.48	27.10, 0.46	17.974,0.022
		Uterine cancer	6616	38	35	0.00, 0.9	17.13, 0.93	17.15, 0.95	19.918,0.025
		Ovarian cancer	6616	38	34	-0.02, 0.64	20.47, 0.81	20.70, 0.84	20.301,0.025
	Primary lymphoid and hematopoietic system	Hematopoietic system cancer	6616	38	34	0.00, 0.93	24.84, 0.53	24.84, 0.58	17.308,0.021
		Hodgkin lymphoma	6616	38	35	-0.02, 0.72	31.34, 0.26	31.49, 0.30	17.389,0.022
		Diffuse large B-cell lymphoma	6616	38	34	-0.05, 0.52	20.47, 0.77	20.89, 0.79	17.299,0.021
		Follicular lymphoma	6616	38	33	-0.00, 0.97	30.74, 0.24	30.74, 0.28	19.723,0.024
		Mature T/NK-cell lymphomas	6616	38	35	0.10, 0.18	24.19, 0.62	26.05, 0.57	19.152,0.024
		Lymphoid leukaemia	6616	38	35	0.04, 0.28	28.08, 0.46	29.29, 0.45	20.099,0.026
		Multiple myeloma and malignant plasma cell cancers	6616	38	34	-0.06, 0.08	25.01, 0.57	28.28, 0.45	20.505,0.025
	Male genital organs	Male genital organs cancers	6616	38	35	0.01, 0.52	22.35, 0.72	22.77, 0.74	19.057,0.024
		Prostatic cancer	6616	38	35	0.01, 0.52	22.62, 0.71	23.05, 0.73	19.057,0.024
CD	Others	Breast cancer	2860	104	96	-0.00, 0.76	104.52, 0.16	104.62, 0.17	19.884,0.058
		Glioma	2860	104	101	0.04, 0.28	93.01, 0.57	94.19, 0.56	20.173,0.062
		Brain cancer	2860	104	99	0.00, 0.84	97.09, 0.39	97.13, 0.42	20.15,0.061
		Meningioma	2860	104	101	-0.00, 0.86	99.84, 0.37	99.87, 0.40	20.222,0.062
		Thyroid cancer	2860	104	100	0.01, 0.42	89.58, 0.64	90.23, 0.65	20.396,0.062
		Oral and pharyngeal cancer	2860	104	102	0.03, 0.34	73.16, 0.97	74.09, 0.97	20.326,0.063
	Urinary system	Urinary organs cancer	2860	104	101	-0.01, 0.45	110.78, 0.14	111.43, 0.15	20.304,0.062
		Bladder cancer	2860	104	96	-0.01, 0.47	83.72, 0.69	84.24, 0.71	20.337,0.059
		Kidney cancer (except renal pelvis)	2860	104	97	-0.01, 0.6	88.62, 0.58	88.89, 0.60	19.478,0.057
	Skin	Skin cancer	2860	104	98	-0.00, 0.69	92.82, 0.49	92.99, 0.51	20.446,0.061
		Melanoma	2860	104	101	0.04, 0.31	83.45, 0.82	84.48, 0.81	20.503,0.063
		Non-melanoma skin cancer	2860	104	98	-0.00, 0.69	93.05, 0.48	93.21, 0.50	20.446,0.061
	Respiratory system and intrathoracic organs	Respiratory system cancers	2860	104	99	0.01, 0.37	101.31, 0.29	102.18, 0.29	20.202,0.061
		Bronchogenic carcinoma and lung cancer	2860	104	97	0.00, 0.68	79.83, 0.81	80.00, 0.83	20.347,0.06
		Non-small cell lung cancer	2860	104	100	-0.00, 0.9	114.42, 0.09	114.44, 0.10	20.404,0.062
		Small cell lung cancer	2860	104	100	-0.01, 0.72	86.60, 0.72	86.73, 0.74	20.048,0.061
	Digestive system	Gastric carcinoma	2860	104	103	-0.02, 0.27	80.06, 0.91	81.31, 0.90	20.232,0.063
		Esophagus cancer	2860	104	102	-0.05, 0.06	95.57, 0.52	99.27, 0.45	20.183,0.062
		Liver cancer	2860	104	98	0.00, 0.91	95.98, 0.40	96.00, 0.42	19.997,0.059
		Pancreatic cancer	2860	104	102	0.01, 0.5	94.89, 0.54	95.35, 0.56	20.275,0.063
	Female genital organs	Female genital organs cancers	2860	104	102	-0.00, 0.72	96.28, 0.50	96.41, 0.53	20.352,0.063
		Cervical cancer	2860	104	99	-0.02, 0.11	81.63, 0.81	84.21, 0.78	20.415,0.061
		Uterine cancer	2860	104	101	0.02, 0.14	95.31, 0.50	97.58, 0.46	20.416,0.063
		Ovarian cancer	2860	104	102	-0.01, 0.46	91.09, 0.65	91.65, 0.66	20.063,0.062
	Primary lymphoid and hematopoietic system	Hematopoietic system cancer	2860	104	98	0.01, 0.44	85.09, 0.71	85.69, 0.72	20.571,0.061
		Hodgkin lymphoma	2860	104	101	0.04, 0.07	107.44, 0.20	111.32, 0.15	20.28,0.062
		Diffuse large B-cell lymphoma	2860	104	101	-0.01, 0.75	87.64, 0.72	87.75, 0.74	20.265,0.062
		Follicular lymphoma	2860	104	100	-0.02, 0.17	82.30, 0.82	84.25, 0.80	19.239,0.058
		Mature T/NK-cell lymphomas	2860	104	103	0.03, 0.36	104.92, 0.30	105.84, 0.30	20.232,0.063
		Lymphoid leukaemia	2860	104	101	0.02, 0.26	80.92, 0.86	82.20, 0.86	19.788,0.061
		Multiple myeloma and malignant plasma cell cancers	2860	104	98	-0.01, 0.72	92.18, 0.50	92.32, 0.53	20.521,0.061
	Male genital organs	Male genital organs cancers	2860	104	100	0.00, 0.71	82.23, 0.82	82.37, 0.84	20.459,0.062
		Prostatic cancer	2860	104	100	0.00, 0.7	84.75, 0.77	84.91, 0.78	20.459,0.062

SNP, Single Nucleotide Polymorphism; LD, Linkage Disequilibrium; MR, Mendelian Randomization; IVW, Inverse Variance Weighted; F, ((Sample size - numbers of IVs - 1) / numbers of IVs) * (r^2^ / (1 - r^2^)), r^2^ = 2 * minor allele frequency (MAF) * (1 - MAF) * β * β / SD^2^; IBD, Inflammatory Bowel Disease; UC, Ulcerative Colitis; CD, Crohn's Disease.

### Two-sample MR analysis

Supplementary Table 1 was the analysis results of the potential causal relationship between IBD and parenteral malignancies through three different methods. In brief, Table 3 shows the significant association between IBD and parenteral malignancies. Patients with IBD had higher odds of diffuse large B-cell lymphoma (OR = 1.2450, 95% CI: 1.0311‒1.5034). Among the population in the FinnGen, having UC was associated with higher odds of both non-melanoma skin cancer (OR = 1.0449, 95% CI: 1.0030‒1.0886) and melanoma (OR = 1.0280, 95% CI: 0.9860‒1.0718). Also, having CD was associated with higher odds of both non-melanoma skin cancer (OR = 1.0288, 95% CI: 1.0023‒1.0560) and skin cancer (OR = 1.0287, 95% CI: 1.0022‒1.0559). In addition, these results were relatively robust due to all powers of the IVW method ≥ 98%.

**Table 3:** Association between IBD and parenteral malignancies.

Exposures	Outcomes	IVW		
		OR (95% CI)	p	Power (%)
IBD	FinnGen			
	Diffuse large B-cell lymphoma	1.2450 (1.0311‒1.5034)	0.023	100
UC	**FinnGen**			
	Non-melanoma skin cancer	1.0449 (1.0030‒1.0886)	0.035	100
	Melanoma	1.0280 (0.9860‒1.0718)	0.019	98
	Skin cancer	0.8581 (0.6283‒1.1720)	0.336	
	**UKB**			
	Non-melanoma skin cancer	1.0034 (1.0015‒1.0052)	<0.001	8
	Melanoma	1.0003 (0.9998‒1.0008)	0.311	
	Skin cancer	1.0004 (1.0001‒1.0006)	0.007	9
CD	**FinnGen**			
	Non-melanoma skin cancer	1.0288 (1.0023‒1.0560)	0.034	99
	Melanoma	1.0004 (0.7892‒1.2680)	0.998	
	Skin cancer	1.0287 (1.0022‒1.0559)	0.033	99
	**UKB**			
	Non-melanoma skin cancer	1.0017 (1.0001‒1.0033)	0.033	6
	Melanoma	1.0004 (0.9999 ‒1.0008)	0.076	
	Skin cancer	1.0002 (0.9999 ‒1.0005)	0.078	

IBD, Inflammatory Bowel Disease; IVW, Inverse Variance Weighted; OR, Odds Ratio, CI, Confidence Interval; UC, Ulcerative Colitis; UKB, the UK Biobank; CD, Crohn's Disease.

Among the UKB population, patients who had UC or CD both seemed to have higher odds of non-melanoma skin cancer (all p < 0.05), whereas having UC was additionally associated with higher odds of skin cancer (OR = 1.0004, 95% CI: 1.0001‒1.0006). Although the power of results in the UKB population was less than 10%, the authors additionally performed the heterogeneity and pleiotropy tests (Supplementary Table 2 and Supplementary Table 3). The findings suggested that no heterogeneity and pleiotropy were found.

## Discussion

The authors conducted a two-sample MR analysis to investigate the potential causal relationship between IBD and parenteral malignancies. Based on the large-scale summary statistics of independent genetic variants that are closely linked to IBD, the authors found patients with IBD have higher odds of both diffuse large B-cell lymphoma and skin cancers, including non-melanoma skin cancer and melanoma.

In the current study, the authors included multiple systems in extraintestinal manifestations of IBD, such as urinary, respiratory, digestive, genital, and hematopoietic systems. Previous studies have proposed that it is of great importance and urgency to clarify the relationship between IBD and parenteral malignancies. In a recent two-sample MR analysis, Lu et al. demonstrated^15^ that IBD, especially CD, is causally responsible for diffuse large B-cell lymphoma. The present study further proved Lu's results. In a large-sample prospective cohort study among adults from the UKB conducted by Wu et al.^16^ showed that IBD may be associated with an increased risk of overall cancer compared with non-IBD, and an increased risk of digestive cancers, non-melanoma skin cancer, and male genital cancers were observed in patients with IBD. These findings in UKB populations similarly indicated a potential causal relationship of UC with CD and non-melanoma skin cancer. This study used the MR analysis in addition to Wu's research, which is less susceptible to confounding bias than that of traditional observational epidemiological studies, and found no heterogeneity and pleiotropy in the potential causal association between UC and CD and non-melanoma skin cancer. Moreover, Gao et al.^12^ also performed a MR study on causality from IBD to 32 site-specific parenteral malignancies, and revealed that IBD has potential causal associations with oral cavity cancer as well as breast cancer. Unfortunately, although the authors explored these relationships in adults from both the FinnGen and UKB databases, we concluded potential causalities between IBD and diffuse large B-cell lymphoma and skin cancers instead of oral cavity cancer or breast cancer. The possible reasons to explain these differences in results between ours and Gao's may be that due to neither the UKB nor FinnGen databases containing the separate GWAS of oral cavity and pharynx cancers, Gao chose a previous conducted GWAS as the discovery cohort.^17^ The authors used the combined data on oral cavity and pharynx cancer, which may limit the true effect of IBD on the occurrence of oral cavity cancers. Also, data sources for cancer in Gao's study were from different databases (more than 5 databases) which may have caused the population heterogeneity. In the present study, the authors additionally calculated the power of the IVW method (powers of results in FinnGen population ≥ 98%) as well as performed the heterogeneity and pleiotropy tests on results among the UKB population (all p > 0.05). Since the relative robustness of the present findings, the authors may supplement Gao's results that IBD has a potential causal relationship with diffuse large B-cell lymphoma, melanoma, and non-melanoma skin cancer.

The underlying mechanisms of causal associations between IBD and diffuse large B-cell lymphoma and skin cancers are unclear, and speculations from previous studies are summarized as follows. Immune dysregulation as well as chronic inflammatory response play significant roles in IBD's development and progression.^18^^,^^19^ In autoimmunity and inflammation conditions, B cells are exposed to multiple types of antigens, which can activate B-cell receptor signaling pathways and also sustain response, proliferation, and clonal amplification. Besides, an increased risk of inherent genetic instability events in lymphocytes during B-cell maturation may in turn lead to malignant lymphoma ultimately development.^20^^,^^21^ IBD has been reported to be associated with an increased risk of melanoma, independent of the use of biological therapy.^22^ The risk of melanoma increased among patients with both CC (RR = 1.80) and UC (RR = 1.23). Also, patients with IBD, especially those who receive thiopurines, are at risk for non-melanoma skin cancer.^23^ The potential mechanisms of the causal relationship from IBD to melanoma and non-melanoma skin cancer are possibly related to epigenetic alterations, such as DNA methylation, histone hyperacetylation, and non-coding RNA in the disease progression,^24^ as well as the disturbance of the microbiota balance in IBD, for example the *Staphylococcus aureus, Streptococcus pyogenes, Pseudomonas aeruginosa strains, β-Human papillomavirus genotypes*, may contribute to the induction of a state of chronic self-maintaining inflammation, leading to skin cancers.^25^ Nevertheless, the specific mechanism that IBD results in skin cancers needs to be further verified.

As mentioned, MR may be a superior research design to confirm the causality from potential risk factors to diseases of interest compared with traditional observational studies. By exploring the potential causal relationship between IBD and parenteral malignancies, these results may facilitate the recommendation of public health policies as well as clinical interventions that effectively reduce the incidence and social burden of parenteral malignancies in patients with IBD. Compared to previous MR studies, statistical analyses in the current study are stricter. However, there are still some limitations in this study. The association between IBD and parenteral malignancies was limited to the European population, which may have possible selection biases, and the results can be generalizable to populations with other races needs further confirmation. Although the authors have made lots of effort to try to prevent IVs from affecting outcomes through confounding factors or other means, it is hard to avoid all confounding factors since carcinogenesis is multifactorial. Therefore, the positive effect of IBD on diffuse large B-cell lymphoma and skin cancers needs to be further validated in randomized controlled trials.

## Conclusion

IBD may have a potential causal association with the risk of diffuse large B-cell lymphoma, melanoma, and non-melanoma skin cancer. Further studies are warranted to elucidate the underlying mechanisms of these causal relationships in patients with IBD.

## Declarations

Ethics approval and consent to participate: Not applicable. Data in the current study are publicly available and de-identified. Each GWAS involved has obtained informed consent from participants and had ethical approval from their respective institutions. Therefore, no ethical approval from the Institutional Review Board (IRB) of The First People's Hospital of Foshan City was required.

## Consent for publication: Not applicable

Availability of data and materials: The datasets used and/or analyzed during the current study are available from the corresponding author on reasonable request.

## Authors’ contributions

(1) Peizhu Su, Zhaotao Li, conceiving and designing the study. (2) Peizhu Su, Yilin Wang, Huiwen Huang, Qinghua Lu, Qinyan Wu, collecting the data. (3) Peizhu Su, Yilin Wang, Huiwen Huang, Qinghua Lu, Qinyan Wu, analyzing and interpreting the data. (4) Peizhu Su, writing the manuscript. (5) Zhaotao Li, Peizhu Su, providing critical revisions that are important for the intellectual content. (6) Peizhu Su, Yilin Wang, Huiwen Huang, Qinghua Lu, Qinyan Wu, Zhaotao Li, approving the final version of the manuscript.

## Funding

This work was supported in part by the Guangdong Medical Research Foundation (No. B2022173) and the Foshan 14th-fifth high-level key specialty construction project (FSGSP145001).

## Conflicts of interest

The authors declare no conflicts of interest.

## Appendix Supplementary materials

Image, application 1
