# Supplementary material for: Association between inflammatory bowel disease and the risk of parenteral malignancies: A two-sample Mendelian randomization study
Source: Clinics (Sao Paulo). 2024 Jun 28;79:100421. doi: 10.1016/j.clinsp.2024.100421 (PMC11260596; doi:10.1016/j.clinsp.2024.100421)
Supplement: Supplementary file 1 [file mmc1.docx]

**CLINICS-D-24-00028_ Supplementary Material**

**Supplementary Table 1** Relationship between IBD and parenteral malignancies.

| **Phenotypes** | **Outcomes** | **Exposures** | **Methods** | **OR (95% CI)** | **p** |
| --- | --- | --- | --- | --- | --- |
| Others | Breast cancer | IBD | MR-Egger | 1.0721 (0.9774‒1.1760) | 0.143 |
|  |  | IBD | Weighted median | 0.9871 (0.9307‒1.0468) | 0.664 |
|  |  | IBD | IVW | 0.9966 (0.9584‒1.0363) | 0.863 |
|  | Glioma | IBD | MR-Egger | 0.9750 (0.4939‒1.9248) | 0.942 |
|  |  | IBD | Weighted median | 0.7090 (0.4573‒1.0990) | 0.124 |
|  |  | IBD | IVW | 0.8112 (0.6103‒1.0782) | 0.150 |
|  | Brain cancer | IBD | MR-Egger | 1.0144 (0.7516‒1.3691) | 0.926 |
|  |  | IBD | Weighted median | 0.9165 (0.7580‒1.1081) | 0.368 |
|  |  | IBD | IVW | 0.9041 (0.7965‒1.0261) | 0.119 |
|  | Meningioma | IBD | MR-Egger | 1.0434 (0.8096‒1.3448) | 0.743 |
|  |  | IBD | Weighted median | 1.0103 (0.8617‒1.1844) | 0.900 |
|  |  | IBD | IVW | 1.0001 (0.8991‒1.1123) | 0.999 |
|  | Thyroid cancer | IBD | MR-Egger | 0.9753 (0.7921‒1.2008) | 0.814 |
|  |  | IBD | Weighted median | 1.1024 (0.9592‒1.2670) | 0.170 |
|  |  | IBD | IVW | 1.0332 (0.9468‒1.1276) | 0.463 |
|  | Oral and pharyngeal cancer | IBD | MR-Egger | 0.9367 (0.5315‒1.6508) | 0.821 |
|  |  | IBD | Weighted median | 1.0509 (0.7266‒1.5200) | 0.792 |
|  |  | IBD | IVW | 1.0871 (0.8573‒1.3786) | 0.491 |
| Urinary organs | Urinary organs cancer | IBD | MR-Egger | 0.9954 (0.8653‒1.1452) | 0.949 |
|  |  | IBD | Weighted median | 0.9835 (0.8967‒1.0788) | 0.725 |
|  |  | IBD | IVW | 0.9741 (0.9184‒1.0331) | 0.381 |
|  | Bladder cancer | IBD | MR-Egger | 1.1230 (0.9229‒1.3665) | 0.249 |
|  |  | IBD | Weighted median | 1.0594 (0.9318‒1.2045) | 0.378 |
|  |  | IBD | IVW | 1.0103 (0.9303‒1.0971) | 0.808 |
|  | Kidney cancer (except renal pelvis) | IBD | MR-Egger | 0.8899 (0.7181‒1.1028) | 0.289 |
|  |  | IBD | Weighted median | 0.9739 (0.8474‒1.1192) | 0.709 |
|  |  | IBD | IVW | 0.9766 (0.8925‒1.0688) | 0.607 |
| Skin | Skin cancer | IBD | MR-Egger | 1.0186 (0.9480‒1.0945) | 0.616 |
|  |  | IBD | Weighted median | 1.0252 (0.9804‒1.0720) | 0.275 |
|  |  | IBD | IVW | 1.0193 (0.9892‒1.0504) | 0.211 |
|  | Melanoma | IBD | MR-Egger | 0.9958 (0.5212‒1.9025) | 0.990 |
|  |  | IBD | Weighted median | 0.9145 (0.6055‒1.3813) | 0.671 |
|  |  | IBD | IVW | 1.0024 (0.7648‒1.3140) | 0.986 |
|  | Non-melanoma skin cancer | IBD | MR-Egger | 1.0185 (0.9479‒1.0944) | 0.617 |
|  |  | IBD | Weighted median | 1.0249 (0.9776‒1.0746) | 0.308 |
|  |  | IBD | IVW | 1.0193 (0.9892‒1.0504) | 0.211 |
| Respiratory system and intrathoracic organs | Respiratory system cancers | IBD | MR-Egger | 0.9781 (0.8434‒1.1342) | 0.770 |
|  |  | IBD | Weighted median | 1.0343 (0.9412‒1.1367) | 0.483 |
|  |  | IBD | IVW | 1.0114 (0.9503‒1.0764) | 0.721 |
|  | Bronchogenic carcinoma and lung cancer | IBD | MR-Egger | 1.0515 (0.8914‒1.2404) | 0.552 |
|  |  | IBD | Weighted median | 1.0050 (0.8987‒1.1238) | 0.930 |
|  |  | IBD | IVW | 1.0160 (0.9479‒1.0889) | 0.654 |
|  | Non-small cell lung cancer | IBD | MR-Egger | 1.1591 (0.9857‒1.3631) | 0.077 |
|  |  | IBD | Weighted median | 1.0242 (0.9217‒1.1381) | 0.656 |
|  |  | IBD | IVW | 1.0293 (0.9613‒1.1021) | 0.407 |
|  | Small cell lung cancer | IBD | MR-Egger | 0.7280 (0.4514‒1.1740) | 0.195 |
|  |  | IBD | Weighted median | 0.8036 (0.5929‒1.0893) | 0.159 |
|  |  | IBD | IVW | 0.8878 (0.7260‒1.0856) | 0.246 |
| Digestive system | Gastric carcinoma | IBD | MR-Egger | 1.0524 (0.8147‒1.3594) | 0.697 |
|  |  | IBD | Weighted median | 1.1059 (0.9418‒1.2985) | 0.219 |
|  |  | IBD | IVW | 1.0384 (0.9332‒1.1555) | 0.489 |
|  | Esophagus cancer | IBD | MR-Egger | 1.1932 (0.7836‒1.8170) | 0.412 |
|  |  | IBD | Weighted median | 1.0608 (0.7972‒1.4116) | 0.685 |
|  |  | IBD | IVW | 1.0185 (0.8536‒1.2152) | 0.839 |
|  | Liver cancer | IBD | MR-Egger | 0.9919 (0.6837‒1.4390) | 0.966 |
|  |  | IBD | Weighted median | 0.9737 (0.7707‒1.2303) | 0.824 |
|  |  | IBD | IVW | 1.0128 (0.8662‒1.1842) | 0.874 |
|  | Pancreatic cancer | IBD | MR-Egger | 1.0445 (0.8043‒1.3564) | 0.745 |
|  |  | IBD | Weighted median | 1.0630 (0.8939‒1.2641) | 0.489 |
|  |  | IBD | IVW | 1.0175 (0.9119‒1.1352) | 0.757 |
| Female genital organs | Female genital organs cancers | IBD | MR-Egger | 0.9301 (0.8348‒1.0363) | 0.191 |
|  |  | IBD | Weighted median | 0.9763 (0.9108‒1.0465) | 0.499 |
|  |  | IBD | IVW | 0.9884 (0.9445‒1.0343) | 0.614 |
|  | Cervical cancer | IBD | MR-Egger | 0.9212 (0.7844‒1.0818) | 0.319 |
|  |  | IBD | Weighted median | 0.9886 (0.8911‒1.0968) | 0.829 |
|  |  | IBD | IVW | 0.9757 (0.9118‒1.0442) | 0.478 |
|  | Uterine cancer | IBD | MR-Egger | 0.9334 (0.7629‒1.1422) | 0.505 |
|  |  | IBD | Weighted median | 0.9503 (0.8400‒1.0751) | 0.418 |
|  |  | IBD | IVW | 0.9866 (0.9064‒1.0738) | 0.754 |
|  | Ovarian cancer | IBD | MR-Egger | 1.0646 (0.8355‒1.3566) | 0.614 |
|  |  | IBD | Weighted median | 1.0621 (0.9011‒1.2518) | 0.473 |
|  |  | IBD | IVW | 0.9977 (0.9013‒1.1044) | 0.965 |
| Primary lymphoid and hematopoietic malignant neoplasms | Hematopoietic system cancer | IBD | MR-Egger | 0.9841 (0.8691‒1.1144) | 0.801 |
|  |  | IBD | Weighted median | 0.9847 (0.9113‒1.0641) | 0.697 |
|  |  | IBD | IVW | 1.0350 (0.9825‒1.0904) | 0.195 |
|  | Hodgkin lymphoma | IBD | MR-Egger | 1.0114 (0.7174‒1.4259) | 0.948 |
|  |  | IBD | Weighted median | 1.0272 (0.8113‒1.3006) | 0.824 |
|  |  | IBD | IVW | 1.0282 (0.8909‒1.1867) | 0.704 |
|  | Diffuse large B-cell lymphoma | IBD | MR-Egger | 1.6904 (1.0759‒2.6557) | 0.025 |
|  |  | IBD | Weighted median | 1.3052 (0.9773‒1.7432) | 0.071 |
|  |  | IBD | IVW | 1.2450 (1.0311‒1.5034) | 0.023 |
|  | Follicular lymphoma | IBD | MR-Egger | 1.0699 (0.7902‒1.4486) | 0.663 |
|  |  | IBD | Weighted median | 1.0248 (0.8394‒1.2512) | 0.810 |
|  |  | IBD | IVW | 0.9522 (0.8389‒1.0808) | 0.449 |
|  | Mature T/NK-cell lymphomas | IBD | MR-Egger | 1.0001 (0.5902‒1.6948) | 1.000 |
|  |  | IBD | Weighted median | 0.9923 (0.7106‒1.3857) | 0.964 |
|  |  | IBD | IVW | 0.8553 (0.6863‒1.0660) | 0.164 |
|  | Lymphoid leukaemia | IBD | MR-Egger | 0.8555 (0.6659‒1.0992) | 0.225 |
|  |  | IBD | Weighted median | 1.0170 (0.8615‒1.2005) | 0.842 |
|  |  | IBD | IVW | 1.0626 (0.9571‒1.1798) | 0.255 |
|  | Multiple myeloma and malignant plasma cell cancers | IBD | MR-Egger | 1.0153 (0.7768‒1.3271) | 0.912 |
|  |  | IBD | Weighted median | 1.0838 (0.9022‒1.3020) | 0.390 |
|  |  | IBD | IVW | 1.0620 (0.9488‒1.1888) | 0.295 |
| Male genital organs | Male genital organs cancers | IBD | MR-Egger | 0.9469 (0.8622‒1.0399) | 0.256 |
|  |  | IBD | Weighted median | 1.0275 (0.9680‒1.0906) | 0.372 |
|  |  | IBD | IVW | 1.0096 (0.9715‒1.0492) | 0.627 |
|  | Prostatic cancer | IBD | MR-Egger | 0.9708 (0.8807‒1.0701) | 0.552 |
|  |  | IBD | Weighted median | 1.0281 (0.9638‒1.0968) | 0.400 |
|  |  | IBD | IVW | 1.0040 (0.9646‒1.0450) | 0.845 |
| Others | Breast cancer | UC | MR-Egger | 0.9489 (0.8116‒1.1094) | 0.516 |
|  |  | UC | Weighted median | 0.9706 (0.9179‒1.0263) | 0.294 |
|  |  | UC | IVW | 0.9723 (0.9304‒1.0160) | 0.210 |
|  | Glioma | UC | MR-Egger | 0.7316 (0.2860‒1.8715) | 0.520 |
|  |  | UC | Weighted median | 0.9106 (0.5849‒1.4176) | 0.678 |
|  |  | UC | IVW | 1.0501 (0.7625‒1.4461) | 0.765 |
|  | Brain cancer | UC | MR-Egger | 0.8494 (0.5599‒1.2886) | 0.449 |
|  |  | UC | Weighted median | 1.0236 (0.8272‒1.2667) | 0.830 |
|  |  | UC | IVW | 0.9859 (0.8526‒1.1401) | 0.848 |
|  | Meningioma | UC | MR-Egger | 0.8925 (0.6260‒1.2725) | 0.535 |
|  |  | UC | Weighted median | 1.0134 (0.8469‒1.2128) | 0.884 |
|  |  | UC | IVW | 1.0522 (0.9301‒1.1903) | 0.419 |
|  | Thyroid cancer | UC | MR-Egger | 0.9620 (0.7197‒1.2858) | 0.795 |
|  |  | UC | Weighted median | 1.0325 (0.8977‒1.1875) | 0.654 |
|  |  | UC | IVW | 1.0202 (0.9239‒1.1266) | 0.692 |
|  | Oral and pharyngeal cancer | UC | MR-Egger | 0.8293 (0.3748‒1.8354) | 0.648 |
|  |  | UC | Weighted median | 0.9966 (0.6655‒1.4924) | 0.987 |
|  |  | UC | IVW | 1.0594 (0.8092‒1.3870) | 0.675 |
| Urinary organs | Urinary organs cancer | UC | MR-Egger | 1.0736 (0.8826‒1.3059) | 0.483 |
|  |  | UC | Weighted median | 0.9443 (0.8558‒1.0419) | 0.253 |
|  |  | UC | IVW | 0.9604 (0.8983‒1.0268) | 0.237 |
|  | Bladder cancer | UC | MR-Egger | 1.1521 (0.8784‒1.5111) | 0.315 |
|  |  | UC | Weighted median | 1.0035 (0.8838‒1.1394) | 0.957 |
|  |  | UC | IVW | 0.9878 (0.9005‒1.0836) | 0.795 |
|  | Kidney cancer (except renal pelvis) | UC | MR-Egger | 0.9846 (0.7176‒1.3510) | 0.924 |
|  |  | UC | Weighted median | 0.9410 (0.8098‒1.0933) | 0.427 |
|  |  | UC | IVW | 0.9707 (0.8730‒1.0793) | 0.582 |
| Skin | Skin cancer | UC | MR-Egger | 1.1301 (0.9953‒1.2832) | 0.070 |
|  |  | UC | Weighted median | 1.0134 (0.9622‒1.0674) | 0.614 |
|  |  | UC | IVW | 0.9931 (0.9575‒1.0301) | 0.711 |
|  | Melanoma | UC | MR-Egger | 1.2660 (0.5117‒3.1323) | 0.614 |
|  |  | UC | Weighted median | 0.9573 (0.6123‒1.4967) | 0.848 |
|  |  | UC | IVW | 0.7777 (0.5713‒1.0587) | 0.110 |
|  | Non-melanoma skin cancer | UC | MR-Egger | 1.1293 (0.9946‒1.2824) | 0.072 |
|  |  | UC | Weighted median | 1.0119 (0.9607‒1.0659) | 0.655 |
|  |  | UC | IVW | 0.9929 (0.9572‒1.0298) | 0.701 |
| Respiratory system and intrathoracic organs | Respiratory system cancers | UC | MR-Egger | 0.9784 (0.8005‒1.1958) | 0.833 |
|  |  | UC | Weighted median | 0.9486 (0.8546‒1.0530) | 0.322 |
|  |  | UC | IVW | 0.9624 (0.8979‒1.0315) | 0.278 |
|  | Bronchogenic carcinoma and lung cancer | UC | MR-Egger | 1.0346 (0.8216‒1.3028) | 0.774 |
|  |  | UC | Weighted median | 0.9432 (0.8409‒1.0579) | 0.318 |
|  |  | UC | IVW | 0.9514 (0.8798‒1.0289) | 0.212 |
|  | Non-small cell lung cancer | UC | MR-Egger | 1.1785 (0.9410‒1.4760) | 0.164 |
|  |  | UC | Weighted median | 0.9893 (0.8774‒1.1156) | 0.861 |
|  |  | UC | IVW | 0.9837 (0.9098‒1.0635) | 0.679 |
|  | Small cell lung cancer | UC | MR-Egger | 0.9738 (0.4463‒2.1246) | 0.947 |
|  |  | UC | Weighted median | 0.8693 (0.6304‒1.1987) | 0.393 |
|  |  | UC | IVW | 0.8897 (0.7024‒1.1269) | 0.332 |
| Digestive system | Gastric carcinoma | UC | MR-Egger | 1.0457 (0.7265‒1.5052) | 0.812 |
|  |  | UC | Weighted median | 0.9507 (0.7889‒1.1456) | 0.595 |
|  |  | UC | IVW | 1.0620 (0.9398‒1.2002) | 0.335 |
|  | Esophagus cancer | UC | MR-Egger | 1.3607 (0.6979‒2.6526) | 0.374 |
|  |  | UC | Weighted median | 0.8648 (0.6324‒1.1827) | 0.363 |
|  |  | UC | IVW | 0.9385 (0.7451‒1.1821) | 0.590 |
|  | Liver cancer | UC | MR-Egger | 1.3600 (0.8158‒2.2671) | 0.249 |
|  |  | UC | Weighted median | 1.0172 (0.7803‒1.3261) | 0.900 |
|  |  | UC | IVW | 0.9934 (0.8305‒1.1881) | 0.942 |
|  | Pancreatic cancer | UC | MR-Egger | 1.3583 (0.9427‒1.9570) | 0.111 |
|  |  | UC | Weighted median | 1.0620 (0.8802‒1.2813) | 0.530 |
|  |  | UC | IVW | 1.0161 (0.8966‒1.1515) | 0.803 |
| Female genital organs | Female genital organs cancers | UC | MR-Egger | 0.8617 (0.7373‒1.0071) | 0.072 |
|  |  | UC | Weighted median | 0.9465 (0.8799‒1.0182) | 0.140 |
|  |  | UC | IVW | 0.9666 (0.9154‒1.0208) | 0.222 |
|  | Cervical cancer | UC | MR-Egger | 0.8310 (0.6196‒1.1146) | 0.228 |
|  |  | UC | Weighted median | 0.9618 (0.8561‒1.0807) | 0.513 |
|  |  | UC | IVW | 0.9823 (0.9038‒1.0676) | 0.674 |
|  | Uterine cancer | UC | MR-Egger | 0.9932 (0.7502‒1.3150) | 0.962 |
|  |  | UC | Weighted median | 1.0529 (0.9220‒1.2023) | 0.447 |
|  |  | UC | IVW | 1.0102 (0.9170‒1.1129) | 0.837 |
|  | Ovarian cancer | UC | MR-Egger | 1.0848 (0.7723‒1.5236) | 0.643 |
|  |  | UC | Weighted median | 1.0260 (0.8717‒1.2075) | 0.758 |
|  |  | UC | IVW | 1.0033 (0.8937‒1.1262) | 0.956 |
| Primary lymphoid and hematopoietic malignant neoplasms | Hematopoietic system cancer | UC | MR-Egger | 0.9682 (0.7929‒1.1822) | 0.754 |
|  |  | UC | Weighted median | 0.9978 (0.9216‒1.0802) | 0.956 |
|  |  | UC | IVW | 0.9764 (0.9219‒1.0342) | 0.417 |
|  | Hodgkin lymphoma | UC | MR-Egger | 1.2415 (0.6473‒2.3812) | 0.520 |
|  |  | UC | Weighted median | 1.1412 (0.8778‒1.4835) | 0.324 |
|  |  | UC | IVW | 1.1050 (0.9200‒1.3272) | 0.286 |
|  | Diffuse large B-cell lymphoma | UC | MR-Egger | 1.1709 (0.5178‒2.6475) | 0.708 |
|  |  | UC | Weighted median | 0.8882 (0.6465‒1.2203) | 0.464 |
|  |  | UC | IVW | 0.9041 (0.7161‒1.1413) | 0.396 |
|  | Follicular lymphoma | UC | MR-Egger | 1.0241 (0.6643‒1.5788) | 0.915 |
|  |  | UC | Weighted median | 1.0535 (0.8644‒1.2840) | 0.606 |
|  |  | UC | IVW | 1.0171 (0.8774‒1.1790) | 0.822 |
|  | Mature T/NK-cell lymphomas | UC | MR-Egger | 0.5237 (0.2497‒1.0985) | 0.098 |
|  |  | UC | Weighted median | 0.8330 (0.5743‒1.2081) | 0.335 |
|  |  | UC | IVW | 0.8493 (0.6578‒1.0966) | 0.210 |
|  | Lymphoid leukaemia | UC | MR-Egger | 0.8319 (0.5863‒1.1804) | 0.311 |
|  |  | UC | Weighted median | 0.9358 (0.7879‒1.1114) | 0.449 |
|  |  | UC | IVW | 1.0004 (0.8872‒1.1279) | 0.995 |
|  | Multiple myeloma and malignant plasma cell cancers | UC | MR-Egger | 1.3575 (0.9388‒1.9629) | 0.116 |
|  |  | UC | Weighted median | 1.0126 (0.8361‒1.2265) | 0.898 |
|  |  | UC | IVW | 0.9865 (0.8681‒1.1211) | 0.835 |
| Male genital organs | Male genital organs cancers | UC | MR-Egger | 0.9637 (0.8493‒1.0934) | 0.571 |
|  |  | UC | Weighted median | 1.0330 (0.9678‒1.1025) | 0.329 |
|  |  | UC | IVW | 1.0019 (0.9590‒1.0468) | 0.931 |
|  | Prostatic cancer | UC | MR-Egger | 0.9603 (0.8437‒1.0931) | 0.545 |
|  |  | UC | Weighted median | 1.0161 (0.9519‒1.0845) | 0.631 |
|  |  | UC | IVW | 1.0002 (0.9563‒1.0462) | 0.991 |
| Others | Breast cancer | CD | MR-Egger | 1.0198 (0.9470‒1.0982) | 0.605 |
|  |  | CD | Weighted median | 0.9859 (0.9360‒1.0385) | 0.591 |
|  |  | CD | IVW | 1.0094 (0.9781‒1.0417) | 0.562 |
|  | Glioma | CD | MR-Egger | 0.7012 (0.3904‒1.2592) | 0.238 |
|  |  | CD | Weighted median | 0.7445 (0.5068‒1.0938) | 0.133 |
|  |  | CD | IVW | 0.9410 (0.7342‒1.2059) | 0.631 |
|  | Brain cancer | CD | MR-Egger | 0.9346 (0.7159‒1.2202) | 0.620 |
|  |  | CD | Weighted median | 0.9248 (0.7761‒1.1022) | 0.383 |
|  |  | CD | IVW | 0.9581 (0.8561‒1.0723) | 0.456 |
|  | Meningioma | CD | MR-Egger | 1.0094 (0.8063‒1.2636) | 0.935 |
|  |  | CD | Weighted median | 1.0219 (0.8780‒1.1893) | 0.780 |
|  |  | CD | IVW | 0.9906 (0.9009‒1.0891) | 0.844 |
|  | Thyroid cancer | CD | MR-Egger | 0.9034 (0.7536‒1.0831) | 0.275 |
|  |  | CD | Weighted median | 1.0197 (0.9029‒1.1516) | 0.753 |
|  |  | CD | IVW | 0.9663 (0.8950‒1.0433) | 0.381 |
|  | Oral and pharyngeal cancer | CD | MR-Egger | 0.8214 (0.5029‒1.3418) | 0.434 |
|  |  | CD | Weighted median | 1.0584 (0.7608‒1.4722) | 0.736 |
|  |  | CD | IVW | 1.0225 (0.8315‒1.2572) | 0.833 |
| Urinary organs | Urinary organs cancer | CD | MR-Egger | 1.0406 (0.9126‒1.1866) | 0.554 |
|  |  | CD | Weighted median | 0.9991 (0.9156‒1.0903) | 0.985 |
|  |  | CD | IVW | 0.9941 (0.9406‒1.0505) | 0.832 |
|  | Bladder cancer | CD | MR-Egger | 1.0226 (0.8620‒1.2131) | 0.798 |
|  |  | CD | Weighted median | 0.9634 (0.8599‒1.0793) | 0.520 |
|  |  | CD | IVW | 0.9660 (0.8981‒1.0389) | 0.351 |
|  | Kidney cancer (except renal pelvis) | CD | MR-Egger | 1.0913 (0.9030‒1.3189) | 0.368 |
|  |  | CD | Weighted median | 1.0630 (0.9247‒1.2220) | 0.390 |
|  |  | CD | IVW | 1.0425 (0.9626‒1.1289) | 0.306 |
| Skin | Skin cancer | CD | MR-Egger | 1.0405 (0.9786‒1.1062) | 0.207 |
|  |  | CD | Weighted median | 1.0315 (0.9909‒1.0739) | 0.130 |
|  |  | CD | IVW | 1.0287 (1.0022‒1.0559) | 0.034 |
|  | Melanoma | CD | MR-Egger | 0.7672 (0.4357‒1.3510) | 0.361 |
|  |  | CD | Weighted median | 0.9879 (0.6861‒1.4225) | 0.948 |
|  |  | CD | IVW | 1.0004 (0.7892‒1.2680) | 0.998 |
|  | Non-melanoma skin cancer | CD | MR-Egger | 1.0403 (0.9785‒1.1060) | 0.209 |
|  |  | CD | Weighted median | 1.0323 (0.9904‒1.0760) | 0.132 |
|  |  | CD | IVW | 1.0288 (1.0023‒1.0560) | 0.033 |
| Respiratory system and intrathoracic organs | Respiratory system cancers | CD | MR-Egger | 0.9508 (0.8353‒1.0822) | 0.447 |
|  |  | CD | Weighted median | 1.0187 (0.9368‒1.1076) | 0.665 |
|  |  | CD | IVW | 1.0033 (0.9492‒1.0605) | 0.906 |
|  | Bronchogenic carcinoma and lung cancer | CD | MR-Egger | 0.9718 (0.8458‒1.1166) | 0.687 |
|  |  | CD | Weighted median | 0.9673 (0.8806‒1.0625) | 0.487 |
|  |  | CD | IVW | 0.9983 (0.9404‒1.0597) | 0.955 |
|  | Non-small cell lung cancer | CD | MR-Egger | 1.0273 (0.8807‒1.1985) | 0.732 |
|  |  | CD | Weighted median | 0.9838 (0.8943‒1.0822) | 0.737 |
|  |  | CD | IVW | 1.0181 (0.9541‒1.0865) | 0.588 |
|  | Small cell lung cancer | CD | MR-Egger | 1.1765 (0.6952‒1.9910) | 0.546 |
|  |  | CD | Weighted median | 1.0811 (0.8087‒1.4453) | 0.599 |
|  |  | CD | IVW | 1.0751 (0.8932‒1.2939) | 0.444 |
| Digestive system | Gastric carcinoma | CD | MR-Egger | 1.1853 (0.9493‒1.4800) | 0.137 |
|  |  | CD | Weighted median | 1.0853 (0.9442‒1.2475) | 0.249 |
|  |  | CD | IVW | 1.0570 (0.9627‒1.1604) | 0.245 |
|  | Esophagus cancer | CD | MR-Egger | 1.3425 (0.9326‒1.9325) | 0.116 |
|  |  | CD | Weighted median | 1.0889 (0.8528‒1.3905) | 0.495 |
|  |  | CD | IVW | 0.9708 (0.8313‒1.1338) | 0.708 |
|  | Liver cancer | CD | MR-Egger | 1.0355 (0.7471‒1.4351) | 0.835 |
|  |  | CD | Weighted median | 1.0820 (0.8764‒1.3358) | 0.464 |
|  |  | CD | IVW | 1.0541 (0.9176‒1.2109) | 0.456 |
|  | Pancreatic cancer | CD | MR-Egger | 0.9166 (0.7286‒1.1530) | 0.459 |
|  |  | CD | Weighted median | 0.9712 (0.8375‒1.1262) | 0.699 |
|  |  | CD | IVW | 0.9846 (0.8940‒1.0844) | 0.753 |
| Female genital organs | Female genital organs cancers | CD | MR-Egger | 0.9834 (0.8950‒1.0805) | 0.728 |
|  |  | CD | Weighted median | 0.9555 (0.8957‒1.0193) | 0.168 |
|  |  | CD | IVW | 0.9682 (0.9307‒1.0072) | 0.108 |
|  | Cervical cancer | CD | MR-Egger | 1.0615 (0.9238‒1.2198) | 0.402 |
|  |  | CD | Weighted median | 0.9753 (0.8885‒1.0707) | 0.600 |
|  |  | CD | IVW | 0.9575 (0.9025‒1.0159) | 0.150 |
|  | Uterine cancer | CD | MR-Egger | 0.8373 (0.7034‒0.9966) | 0.049 |
|  |  | CD | Weighted median | 0.9184 (0.8142‒1.0359) | 0.166 |
|  |  | CD | IVW | 0.9452 (0.8778‒1.0178) | 0.136 |
|  | Ovarian cancer | CD | MR-Egger | 1.0460 (0.8487‒1.2892) | 0.674 |
|  |  | CD | Weighted median | 0.9977 (0.8604‒1.1569) | 0.976 |
|  |  | CD | IVW | 0.9731 (0.8907‒1.0631) | 0.546 |
| Primary lymphoid and hematopoietic malignant neoplasms | Hematopoietic system cancer | CD | MR-Egger | 1.0036 (0.9121‒1.1044) | 0.941 |
|  |  | CD | Weighted median | 0.9972 (0.9321‒1.0668) | 0.935 |
|  |  | CD | IVW | 1.0385 (0.9969‒1.0818) | 0.070 |
|  | Hodgkin lymphoma | CD | MR-Egger | 0.7816 (0.5742‒1.0638) | 0.120 |
|  |  | CD | Weighted median | 0.9959 (0.8229‒1.2053) | 0.966 |
|  |  | CD | IVW | 1.0190 (0.8929‒1.1629) | 0.780 |
|  | Diffuse large B-cell lymphoma | CD | MR-Egger | 1.2089 (0.8220‒1.7779) | 0.337 |
|  |  | CD | Weighted median | 1.1655 (0.9040‒1.5026) | 0.237 |
|  |  | CD | IVW | 1.1426 (0.9704‒1.3454) | 0.110 |
|  | Follicular lymphoma | CD | MR-Egger | 1.1336 (0.8845‒1.4527) | 0.324 |
|  |  | CD | Weighted median | 1.0624 (0.8947‒1.2615) | 0.490 |
|  |  | CD | IVW | 0.9664 (0.8693‒1.0742) | 0.526 |
|  | Mature T/NK-cell lymphomas | CD | MR-Egger | 0.7484 (0.4681‒1.1965) | 0.229 |
|  |  | CD | Weighted median | 0.9701 (0.7163‒1.3138) | 0.844 |
|  |  | CD | IVW | 0.9153 (0.7515‒1.1148) | 0.379 |
|  | Lymphoid leukaemia | CD | MR-Egger | 0.8913 (0.7153‒1.1105) | 0.307 |
|  |  | CD | Weighted median | 1.0203 (0.8850‒1.1763) | 0.782 |
|  |  | CD | IVW | 0.9999 (0.9110‒1.0975) | 0.998 |
|  | Multiple myeloma and malignant plasma cell cancers | CD | MR-Egger | 1.0985 (0.8709‒1.3856) | 0.430 |
|  |  | CD | Weighted median | 1.0809 (0.9208‒1.2687) | 0.341 |
|  |  | CD | IVW | 1.0562 (0.9574‒1.1651) | 0.275 |
| Male genital organs | Male genital organs cancers | CD | MR-Egger | 1.0136 (0.9365‒1.0970) | 0.739 |
|  |  | CD | Weighted median | 1.0238 (0.9734‒1.0767) | 0.361 |
|  |  | CD | IVW | 1.0276 (0.9941‒1.0623) | 0.107 |
|  | Prostatic cancer | CD | MR-Egger | 1.0126 (0.9338‒1.0981) | 0.763 |
|  |  | CD | Weighted median | 1.0310 (0.9778‒1.0871) | 0.259 |
|  |  | CD | IVW | 1.0276 (0.9932‒1.0631) | 0.117 |

IBD, Inflammatory Bowel Disease; OR, Odds Ratio; CI, Confidence Interval; MR, Mendelian Randomization; IVW, Inverse Variance Weighted; UC, Ulcerative Colitis; CD, Crohn's Disease.

**Supplementary Table 2** Heterogeneity test of association between IBD and parenteral malignancies in UKB population.

| **Exposures** | **Outcomes** | **MR-Egger intercept** | **p** |
| --- | --- | --- | --- |
| IBD | Bronchogenic carcinoma and lung cancer | 0.0000 | 0.6150 |
|  | Lymphoid leukaemia | 0.0000 | 0.7476 |
|  | Multiple myeloma and malignant plasma cell cancers | 0.0000 | 0.7869 |
|  | Non-melanoma skin cancer | 0.0000 | 0.9436 |
|  | Ovarian cancer | 0.0000 | 0.5592 |
|  | Meningioma | -0.0001 | 0.2931 |
|  | Skin cancer | 0.0000 | 0.7764 |
|  | Kidney cancer (except renal pelvis) | 0.0000 | 0.1972 |
|  | Breast cancer | 0.0000 | 0.3876 |
|  | Ovarian cancer | 0.0000 | 0.4813 |
|  | Prostatic cancer | 0.0000 | 0.9641 |
|  | Bladder cancer | 0.0000 | 0.1287 |
|  | Cervical cancer | 0.0000 | 0.9778 |
|  | Female genital organs cancers | 0.0000 | 0.4639 |
|  | Male genital organs cancers | 0.0000 | 0.3782 |
|  | Uterine cancer | 0.0000 | 0.5928 |
|  | Lymphoid leukaemia | 0.0000 | 0.3597 |
|  | Respiratory system cancers | 0.0000 | 0.6564 |
|  | Urinary organs cancer | 0.0000 | 0.9361 |
|  | Bronchogenic carcinoma and lung cancer | 0.0000 | 0.4696 |
|  | Bronchogenic carcinoma and lung cancer | 0.0000 | 0.7664 |
| UC | Bronchogenic carcinoma and lung cancer | 0.0002 | 0.2536 |
|  | Lymphoid leukaemia | 0.0000 | 0.7170 |
|  | Multiple myeloma and malignant plasma cell cancers | 0.0000 | 0.9411 |
|  | Non-melanoma skin cancer | 0.0000 | 0.7353 |
|  | Ovarian cancer | 0.0002 | 0.0572 |
|  | Meningioma | -0.0001 | 0.5164 |
|  | Skin cancer | -0.0001 | 0.8009 |
|  | Kidney cancer (except renal pelvis) | -0.0001 | 0.5254 |
|  | Breast cancer | 0.0000 | 0.7511 |
|  | Ovarian cancer | -0.0001 | 0.2737 |
|  | Prostatic cancer | 0.0000 | 0.7432 |
|  | Bladder cancer | 0.0001 | 0.1500 |
|  | Uterine cancer | -0.0001 | 0.1104 |
|  | Female genital organs cancers | 0.0001 | 0.4382 |
|  | Male genital organs cancers | -0.0001 | 0.2386 |
|  | Uterine cancer | 0.0000 | 0.8139 |
|  | Lymphoid leukaemia | 0.0000 | 0.7474 |
|  | Respiratory system cancers | 0.0000 | 0.7400 |
|  | Urinary organs cancer | 0.0000 | 0.8037 |
|  | Bronchogenic carcinoma and lung cancer | -0.0001 | 0.6368 |
|  | Bronchogenic carcinoma and lung cancer | 0.0002 | 0.3482 |
| CD | Bronchogenic carcinoma and lung cancer | 0.0001 | 0.3760 |
|  | Lymphoid leukaemia | 0.0000 | 0.9838 |
|  | Multiple myeloma and malignant plasma cell cancers | 0.0000 | 0.4537 |
|  | Non-melanoma skin cancer | 0.0000 | 0.7490 |
|  | Ovarian cancer | 0.0000 | 0.9483 |
|  | Meningioma | 0.0000 | 0.7680 |
|  | Skin cancer | 0.0002 | 0.1997 |
|  | Kidney cancer (except renal pelvis) | -0.0001 | 0.0464 |
|  | Breast cancer | -0.0001 | 0.0421 |
|  | Ovarian cancer | 0.0000 | 0.4002 |
|  | Prostatic cancer | 0.0000 | 0.8791 |
|  | Bladder cancer | 0.0000 | 0.3309 |
|  | Uterine cancer | 0.0000 | 0.6113 |
|  | Female genital organs cancers | 0.0000 | 0.5389 |
|  | Male genital organs cancers | -0.0001 | 0.1661 |
|  | Uterine cancer | 0.0000 | 0.6460 |
|  | Lymphoid leukaemia | -0.0001 | 0.1847 |
|  | Respiratory system cancers | 0.0001 | 0.2501 |
|  | Urinary organs cancer | 0.0000 | 0.4839 |
|  | Bronchogenic carcinoma and lung cancer | 0.0000 | 0.8399 |
|  | Bronchogenic carcinoma and lung cancer | 0.0001 | 0.2134 |

IBD, Inflammatory Bowel Disease; UKB, the UK Biobank; MR, Mendelian Randomization; UC, Ulcerative Colitis; CD, Crohn's Disease.

**Supplementary Table 3** Pleiotropy test of association between IBD and parenteral malignancies in UKB population.

| **Exposures** | **Outcomes** | **MR-Egger** | **p** | **IVW** | **p** |
| --- | --- | --- | --- | --- | --- |
| IBD | Bronchogenic carcinoma and lung cancer | 73.847 | 0.981 | 74.101 | 0.983 |
|  | Lymphoid leukaemia | 80.832 | 0.983 | 80.937 | 0.986 |
|  | Multiple myeloma and malignant plasma cell cancers | 40.540 | 0.940 | 40.614 | 0.950 |
|  | Non-melanoma skin cancer | 44.790 | 0.941 | 44.795 | 0.951 |
|  | Ovarian cancer | 59.926 | 0.864 | 60.270 | 0.875 |
|  | Meningioma | 92.988 | 0.878 | 94.103 | 0.875 |
|  | Skin cancer | 76.066 | 0.793 | 76.147 | 0.812 |
|  | Kidney cancer (except renal pelvis) | 83.458 | 0.980 | 85.141 | 0.977 |
|  | Breast cancer | 80.288 | 0.988 | 81.040 | 0.988 |
|  | Ovarian cancer | 103.085 | 0.759 | 103.584 | 0.769 |
|  | Prostatic cancer | 47.702 | 0.854 | 47.704 | 0.874 |
|  | Bladder cancer | 97.432 | 0.817 | 99.775 | 0.789 |
|  | Cervical cancer | 91.921 | 0.936 | 91.922 | 0.944 |
|  | Female genital organs cancers | 85.068 | 0.900 | 85.608 | 0.905 |
|  | Male genital organs cancers | 78.915 | 0.991 | 79.698 | 0.991 |
|  | Uterine cancer | 80.725 | 0.984 | 81.013 | 0.985 |
|  | Lymphoid leukaemia | 72.460 | 1.000 | 73.305 | 1.000 |
|  | Respiratory system cancers | 94.245 | 0.931 | 94.445 | 0.938 |
|  | Urinary organs cancer | 84.161 | 0.779 | 84.168 | 0.800 |
|  | Bronchogenic carcinoma and lung cancer | 107.390 | 0.727 | 107.916 | 0.736 |
|  | Bronchogenic carcinoma and lung cancer | 104.410 | 0.706 | 104.499 | 0.727 |
| UC | Bronchogenic carcinoma and lung cancer | 29.152 | 0.304 | 30.680 | 0.285 |
|  | Lymphoid leukaemia | 26.877 | 0.470 | 27.011 | 0.518 |
|  | Multiple myeloma and malignant plasma cell cancers | 12.951 | 0.530 | 12.957 | 0.606 |
|  | Non-melanoma skin cancer | 11.953 | 0.803 | 12.072 | 0.844 |
|  | Ovarian cancer | 11.701 | 0.862 | 15.829 | 0.669 |
|  | Meningioma | 24.276 | 0.560 | 24.708 | 0.591 |
|  | Skin cancer | 26.430 | 0.332 | 26.502 | 0.381 |
|  | Kidney cancer (except renal pelvis) | 16.958 | 0.949 | 17.372 | 0.956 |
|  | Breast cancer | 23.065 | 0.774 | 23.167 | 0.808 |
|  | Ovarian cancer | 25.112 | 0.622 | 26.358 | 0.606 |
|  | Prostatic cancer | 9.440 | 0.853 | 9.552 | 0.889 |
|  | Bladder cancer | 22.358 | 0.764 | 24.549 | 0.701 |
|  | Uterine cancer | 23.908 | 0.686 | 26.626 | 0.592 |
|  | Female genital organs cancers | 27.427 | 0.238 | 28.169 | 0.253 |
|  | Male genital organs cancers | 14.737 | 0.962 | 16.192 | 0.949 |
|  | Uterine cancer | 15.395 | 0.932 | 15.452 | 0.949 |
|  | Lymphoid leukaemia | 16.519 | 0.957 | 16.625 | 0.968 |
|  | Respiratory system cancers | 20.155 | 0.888 | 20.267 | 0.909 |
|  | Urinary organs cancer | 20.390 | 0.434 | 20.455 | 0.493 |
|  | Bronchogenic carcinoma and lung cancer | 27.894 | 0.470 | 28.121 | 0.511 |
|  | Bronchogenic carcinoma and lung cancer | 25.741 | 0.533 | 26.652 | 0.537 |
| CD | Bronchogenic carcinoma and lung cancer | 88.490 | 0.555 | 89.282 | 0.561 |
|  | Lymphoid leukaemia | 61.049 | 0.992 | 61.050 | 0.993 |
|  | Multiple myeloma and malignant plasma cell cancers | 40.161 | 0.552 | 40.733 | 0.570 |
|  | Non-melanoma skin cancer | 43.906 | 0.476 | 44.009 | 0.514 |
|  | Ovarian cancer | 59.639 | 0.345 | 59.644 | 0.380 |
|  | Meningioma | 81.873 | 0.717 | 81.961 | 0.740 |
|  | Skin cancer | 56.132 | 0.847 | 57.809 | 0.829 |
|  | Kidney cancer (except renal pelvis) | 55.790 | 0.998 | 59.870 | 0.995 |
|  | Breast cancer | 59.111 | 0.988 | 63.367 | 0.973 |
|  | Ovarian cancer | 88.950 | 0.600 | 89.664 | 0.607 |
|  | Prostatic cancer | 27.627 | 0.967 | 27.650 | 0.974 |
|  | Bladder cancer | 65.668 | 0.950 | 66.624 | 0.949 |
|  | Uterine cancer | 71.069 | 0.940 | 71.329 | 0.946 |
|  | Female genital organs cancers | 59.501 | 0.976 | 59.881 | 0.978 |
|  | Male genital organs cancers | 74.494 | 0.895 | 76.443 | 0.879 |
|  | Uterine cancer | 78.761 | 0.795 | 78.974 | 0.812 |
|  | Lymphoid leukaemia | 80.963 | 0.788 | 82.749 | 0.768 |
|  | Respiratory system cancers | 70.908 | 0.969 | 72.247 | 0.966 |
|  | Urinary organs cancer | 57.054 | 0.901 | 57.550 | 0.908 |
|  | Bronchogenic carcinoma and lung cancer | 70.603 | 0.900 | 70.644 | 0.912 |
|  | Bronchogenic carcinoma and lung cancer | 82.713 | 0.639 | 74.101 | 0.983 |

IBD, Inflammatory Bowel Disease; UKB, the UK Biobank; MR, Mendelian Randomization; IVW, Inverse Variance Weighted; UC, Ulcerative Colitis; CD, Crohn's Disease.
